# Romantic relationship breakup: An experimental model to study effects of stress on depression (-like) symptoms

**DOI:** 10.1371/journal.pone.0217320

**Published:** 2019-05-31

**Authors:** Anne M. Verhallen, Remco J. Renken, Jan-Bernard C. Marsman, Gert J. ter Horst

**Affiliations:** University of Groningen, University Medical Center Groningen, Department of Biomedical Sciences of Cells & Systems, Cognitive Neuroscience Center, Groningen, the Netherlands; University of Lleida, SPAIN

## Abstract

The occurrence of a stressful event is considered to increase the risk of developing depression. In the present study we explore whether the breakup of a romantic relationship can be used as an experimental model to study a depression-like state during a period of stress in individuals without a psychiatric disorder. The primary aim of our study was to investigate: 1) whether individuals with a recent romantic relationship breakup (‘‘heartbreak”) demonstrate symptoms of depression, 2) how to describe heartbreak characteristics based on data from a comprehensive questionnaire battery, and 3) whether this description can capture severity of depression symptoms. Secondary, we were interested in gender differences with regard to the above study objectives. Subjects who have experienced a relationship breakup in the preceding six months (*N* = 71) or are in a romantic relationship (*N* = 46) participated in our study. A questionnaire battery was administered to acquire information related to depression, mood, the breakup and (former) relationship. Principal Component Analysis with Procrustes bootstrapping was performed to extract components from the questionnaire data. Even though our sample of individuals who recently have experienced a relationship breakup can be on average considered non-depressed, group-level depression scores were elevated compared to individuals in a relationship (*p* = .001) and 26.8% reported symptoms corresponding to mild, moderate or severe depression. We described heartbreak by two principal components interpreted as ‘‘sudden loss” and ‘‘lack of positive affect”, respectively. Highly significant correlations between the component scores and depression scores were found (*p* < .001 and *p* < .001, respectively), although these correlations differed between the genders. Based on these findings, we propose that the experience of a romantic relationship breakup is a viable experimental model to examine symptoms of depression in individuals without a psychiatric disorder. This way, stress-related coping and depression vulnerability can be studied in further research.

## Introduction

Stressful life-events are considered to be risk factors for the development of depression[1]. Kendler et al.[2] investigated the interplay between stressful events, genetic predisposition and depression among female twins and found that both heredity and occurrence of stressful events contributed to the onset of depressive episodes independently. Especially events with a high impact, such as death of a close family member and divorce, elevated the probability of developing a depressive episode[2], although the majority of people do not develop a depressive episode following the experience of an upsetting event. Hence, research focusing on stressful and emotionally upsetting events can give valuable insights into individual differences regarding stress-related coping and the link between stress and depression.

In this study we set out to investigate mood and depression symptoms during a period of stress in a population without a psychiatric disorder. More precisely, we explore whether the breakup of a romantic relationship can be used as an experimental model to study a depression-like state. Previous research already showed that the breakup of a romantic relationship can be seen as an emotionally upsetting event that can lead to multiple symptoms related to sadness, grief and depression and even can result in an increased risk of developing a depressive episode[3–6]. In a university student sample, severe breakup distress, measured with a questionnaire concerning symptoms of grief, was accompanied by feelings of betray and rejection, depression symptoms, anxiety symptoms, intrusive thoughts about the ex-partner and sleep disturbances[3]. The elapsed time since the breakup, self-reported quality of the former relationship, feelings of betray and depression scores especially predicted the severity of breakup distress[3]. Additionally, women reported higher breakup distress scores compared to men in that study[3]. In a study of Stoessel et al.[4], all of the subjects with a relationship breakup in the preceding six months and experiencing feelings of sadness about the breakup reported symptoms corresponding to clinical depression. In women with a breakup in the preceding four months, high levels of complicated grief (extreme symptoms of grief interfering with daily life functioning) were present in four of the eight subjects. In addition, a different brain pattern (increased activity in posterior regions such as the cerebellum and decreased activity in anterior regions such as the insula and temporal cortex) was found in these women when ruminating about their ex-partner in comparison with thinking about an acquaintance in a neutral manner[5]. Moreover, epidemiological data indicated an association between the occurrence of a romantic relationship breakup and first onset of major depression in a young population[6].

As it is known that the prevalence of depression is higher in women, we were also interested in differences in depression (-like) symptoms between the genders in our study. For example, data from a United States survey revealed a 1.7 times higher lifetime prevalence of depressive episodes among women[7]. Differences in stress sensitivity between the genders could play a role, as stress paradigms in rodents elucidated different stress responses between males and females[8,9]. Moreover, gender differences with regard to rumination might be involved. It is known that women tend to ruminate more during periods of stress[10]. A ruminative coping strategy was associated with both anxiety and depression symptoms and correlated significantly with occurrence of new depressive episodes in patients with major depressive disorder[11]. In addition, experiencing ruminating thoughts about the loss during grief was found to be related to maladaptive grieving and the development of symptoms of depression[12].

In the present study, we primarily aimed to investigate: 1) whether individuals with a recent romantic relationship breakup (‘‘heartbreak”) demonstrate symptoms of depression, 2) how to describe heartbreak characteristics based on data from a comprehensive questionnaire battery, and 3) whether this description can capture severity of depression symptoms. Secondary, we were interested in gender differences with regard to the above study objectives. To this end, young men and women, either with a recent romantic relationship breakup (the ‘‘heartbreak group”) or in a romantic relationship (the ‘‘relationship group”) participated. The relationship group was included in the study as a reference group with absence of stress resulting from a romantic relationship breakup. We expected a higher severity of depression symptoms in the heartbreak group compared to the relationship group. Given that women are more at risk for developing depression in the general population, we expected a higher severity of depression symptoms among the women in the heartbreak group than the men in the heartbreak group.

## Materials and methods

### Experimental design

Subjects were invited to our laboratory to participate in the study between 2011 and 2013. The experiment comprised a self-report questionnaire battery and fMRI paradigm with a cross-sectional design. fMRI results will be reported elsewhere. Before the start of the study, written informed consent was obtained from every subject. Study procedures were approved by the Medical Ethical Committee of the University Medical Center Groningen and conducted in accordance with the principles of the Declaration of Helsinki. Subjects received a financial compensation for their participation.

### Recruitment strategy

Subjects were recruited by distributing posters around faculty buildings of the University of Groningen and promoting the study using (social) media. Women of the heartbreak group (‘‘heartbroken females”) were recruited using recruitment material with terminology implying that subjects have to suffer from breakup distress to participate. With this recruitment strategy it was not possible to include a sufficient number of male subjects. Therefore, a subsample of the men of the heartbreak group (‘‘heartbroken males”) was recruited using recruitment material referring to the experience of a relationship breakup instead of suffering from breakup distress. Potential subjects could send an email to show their interest in the study and exchange contact information. A telephone intake interview was planned to explain study procedures and check inclusion and exclusion criteria. Additionally, subjects received written information. During the first stage of the study, heartbroken females were pre-selected at the intake interview based on their self-report level of sadness about the breakup on a scale from 1 to 10, because at that time we intended to compare women with contrasting levels of breakup distress. For the results presented in this paper we did not divide the heartbreak group in subgroups based on information obtained at the intake interview.

### Inclusion and exclusion criteria

For both the heartbreak group and the relationship group, subjects had to be between the age of 18 and 26 years, right-handed, Western, heterosexual and Dutch speaking. Female subjects could only participate if they used hormonal contraception and were in the continuation phase on the day of the experiment to minimize possible effects of fluctuating sex hormone levels on our outcome measures. To participate in the heartbreak group, subjects had to have a relationship breakup within the preceding six months and a relationship duration of at least six months. To participate in the relationship group, subjects had to have a relationship duration between 6 and 24 months because we intended to include subjects whose relationship has not yet evolved into a companionate stage[13]. Subjects with a relationship duration shorter than 6 months were excluded due to previous research on increased stress hormone level during these first periods[14]. For both the heartbreak group and the relationship group, subjects with neurological abnormalities, MRI contraindications such as ferromagnetic metal parts in the body, (suspected) pregnancy and claustrophobia, use of psychotropic medication in the last five years, alcohol and/or drug abuse and physical and/or sexual abuse during the relationship (all self-reported) were not allowed to participate in the study. 71 and 46 subjects were included in the heartbreak group and the relationship group, respectively. After the fMRI scanning session, one subject from the heartbreak group was excluded because of substantial brain ventricle abnormalities.

### Questionnaire battery

A self-report questionnaire battery in Dutch was administered to assess psychological and behavioral information of the subjects. Before filling in the questionnaire battery, background information, such as highest completed educational level according to the Dutch educational system and current occupation status, was acquired from the subjects. The heartbreak questionnaire battery consisted of the Major Depression Inventory (MDI) and adjusted versions of the Inventory of Complicated Grief (ICG), Positive and Negative Affect Schedule (PANAS), Perceived Relationship Quality Components Inventory (PRQC), the Hurt-Proneness Scale and the Passionate Love Scale (PLS)[13,15–19]. Additionally, in-house designed questions about the breakup were added to the questionnaire battery, covering aspects such as unexpectedness of the breakup and ruminating thoughts about the ex-partner. For each questionnaire, except the in-house designed questionnaire about the breakup, total scores were calculated and used in further analyses. Cronbach’s alpha scores were calculated for each questionnaire (can be found in S1 Table). The MDI is a 10-item questionnaire to assess symptoms of depression (both core symptoms such as anhedonia and accompanying symptoms such as sleeping difficulties), based on the DSM-IV and ICD-10 diagnostic criteria[15,20]. MDI scores were calculated according to the scoring guidelines for the use of the MDI as a rating scale to measure severity of depression symptoms[20]. MDI scores theoretically range between 0 and 50. Scores between 0 and 20 indicate absence of clinical depression, scores between 21 and 25 correspond to mild depression, scores between 26 and 30 and scores above 31 indicate respectively moderate and severe depression[20]. The ICG is used to assess maladaptive grieving after the loss of a loved one[16]. Similar to the study of Najib et al.[5], the ICG was adjusted so that it was suitable for heartbreak. Thirteen items were extracted from the original 19-item version, by removing items only applicable to death. ICG scores were calculated by summing the scores of the 13 questions and theoretically range between 13 and 130. The PANAS comprises questions about positive and negative affect, representing current mood[17]. PANAS scores were calculated for both the positive affect and negative affect part by summing the scores of the 10 questions and theoretically range between 10 and 100[17]. The PRQC was used to assess self-reported former relationship quality[18]. In this study a 9-item version of the PRQC was extracted from the original 18-item version. PRQC scores were calculated by summing the scores of the 9 questions and theoretically range between 9 and 90. To what extent the subjects were prone to experience hurt feelings was measured with the Hurt-Proneness Scale[19]. Hurt proneness scores were calculated by summing the scores of the 6 questions and theoretically range between 6 and 60. Questions 3, 4 and 6 were reversed scored because high scores characterize low hurt proneness. The PLS can be used to assess intensity of romantic love[13]. A 28-item version of the PLS was extracted from the original 30-item version, by removing two items that are not appropriate for heartbreak. The PLS was filled in exclusively by the subjects who reported to be still in love with their ex-partner at the time of the testing day. PLS scores were not analyzed further. As the PLS was only filled in by the heartbroken subjects who reported to be still in love with their ex-partner, the sample size turned out to be insufficient. All questionnaires, except the MDI, were scored on a 10-point Likert scale, ranging from 1 (‘‘not at all”) to 10 (‘‘extremely”). The MDI was rated on a 6-point Likert scale, ranging from 1 (‘‘not at all”) to 6 (‘‘all the time”). Questions about the relationship breakup were categorical or measured on a 10-point Likert scale. The questionnaire battery of the relationship group consisted of the MDI and adjusted versions of the PANAS, PRQC, Hurt-proneness scale and PLS, similar to the heartbreak group.

### Data analysis

Statistical analyses were conducted with IBM SPSS Statistics version 24 for Windows. A Shapiro-Wilk test for normality was used to check if our data were normally distributed. When data distribution was found to be skewed, non-parametric statistical tests were conducted in further analysis steps.

### Group-level comparisons

Background information and questionnaire data were compared between the heartbreak group and the relationship group using a Mann-Whitney *U* test. Concerning the questionnaire battery of the relationship group, only MDI scores are considered in this manuscript, since we aimed to compare severity of depression symptoms between the heartbreak group and the relationship group.

### Principal component Analysis-varimax

An exploratory Principal Component Analysis (PCA) followed by varimax rotation was performed to extract components representing heartbreak in a data-driven manner. We intended to focus on subjective measures. Consequently, 19 variables were entered into the analysis; ‘‘unexpectedness breakup”, ‘‘feeling rejected”, ‘‘feeling betrayed”, ‘‘feeling angry”, ‘‘feeling sad”, ‘‘feeling disappointed”, ‘‘feeling independent”, ‘‘feeling alone”, ‘‘feeling relieved”, ‘‘feeling hopeful”, ‘‘ruminating thoughts”, ‘‘intrusive thoughts”, ‘‘affection for ex-partner”, ‘‘in love with ex-partner”, ‘‘ICG”, ‘‘PANAS positive”, ‘‘PANAS negative”, ‘‘PRQC” and ‘‘Hurt proneness”. Subjects with missing data were deleted listwise, resulting in a sample size of 69 for the PCA. Principal components were extracted using the correlation matrix, and rotated with varimax with Kaiser normalization[21]. Parallel analysis was performed to determine the optimal number of components[22]. We adapted the online available SPSS script for parallel analysis, written by Brian O’Connor, to our dataset (https://people.ok.ubc.ca/brioconn/nfactors/nfactors.html) [23]. Thousand sets of normally distributed data were randomly generated. For each component an eigenvalue belonging to the original data and an eigenvalue belonging to the 95% confidence interval (CI) of the generated data was computed. Components with an eigenvalue greater than the corresponding eigenvalue derived from random normal data generation were considered as ‘‘components”. Subsequently, a PCA followed by a varimax rotation was performed with a fixed number of components to extract, based on the results of the parallel analysis. The outcome of this combined PCA and varimax rotation, a component matrix, was used in the subsequent analyses.

### Procrustes bootstrapping

A Procrustes bootstrapping PCA was performed to select the component loadings to be interpreted further. Thousand samples of component matrices were generated by resampling with replacement. To this end, we adjusted the online available SPSS script for component analysis with Procrustes bootstrapping from Linda Reichwein Zientek and Bruce Thompson[24]. Note that, just like the original PCA-varimax, components were not normalized row wise. Bootstrapping results were rotated towards a target matrix. The target matrix was constructed by binarizing the component matrix retaining the sign. Variables were assigned 1 or -1 for the component they loaded strongest on and 0 elsewhere. 95% CIs were calculated for each variable across the thousand bootstrap resamples.

### Interpretation components

Only variables with a 95% CI that does not cross zero were interpreted further. Labels were assigned to each component based on the component loadings acquired with the original PCA-varimax.

### Component scores analysis

For each subject, component scores were computed using regression. A Spearman rank test was conducted to see how well the component scores correlate with MDI scores. A Spearman rank test was used to assess the correlation between the component scores, time since breakup and relationship duration. Component scores were compared between men and women with an independent samples t-test. Additionally, Spearman rank correlations between the component scores and MDI scores were calculated for men and women separately.

For all conducted statistical tests, results were considered significant at *p*-value < .05 (uncorrected), two-tailed.

## Results

### Study population

The relationship group consisted of 23 men and 23 women with a relationship duration between 6 and 24 months (*Mdn* = 13.00, *IQR* = 9.00–19.00). Age ranged between 18 and 26 years (*Mdn* = 21.00, *IQR* = 20.00–23.00). The heartbreak group consisted of 33 men and 38 women. Age ranged between 18 and 25 years (*Mdn* = 22.00, *IQR* = 21.00–24.00). Relationship duration prior to the breakup ranged between 6 and 81 months (*Mdn* = 20.00, *IQR* = 13.00–37.00). Time since breakup ranged between 0 and 5 months (*Mdn* = 2.00, *IQR* = 1.00–4.00). In 42.3% of the subjects, the ex-partner decided to break up, whereas in 35.2% the breakup was initiated by the subject and in 22.5% the subject and ex-partner decided together. 70.4% of the subjects reported to still be in touch with their ex-partner. Five subjects (7.0%) reported to have found a new romantic partner. 70.4% reported to still think about their ex-partner on a daily basis and 25.4% experienced physical complaints after the breakup. The heartbreak group was significantly older than the relationship group (*U* = 1241.00, *Z* = -2.21, *p* = .027, *r* = -.20). Additional background information of our study population can be found in S2 Table.

### Severity of depression symptoms

**Fig 1:**
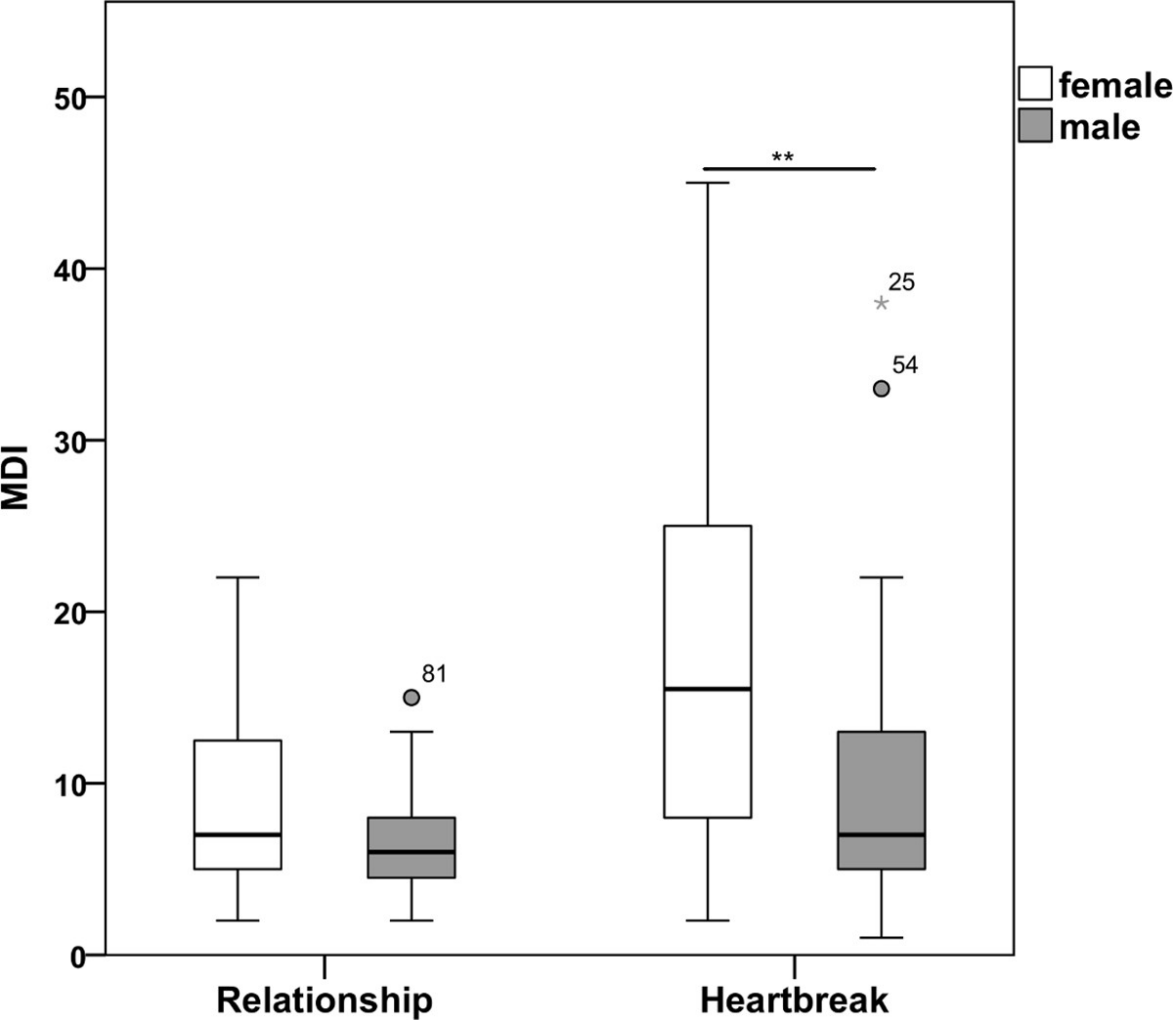
Severity of depression symptoms (MDI scores) in the relationship group and the heartbreak group. Outliers (values that are between Q1-1.5*IQR or Q3+1.5*IQR and Q1-3*IQR or Q3+3*IQR) are indicated with a circle. Extreme outliers (values that are beyond Q1-3*IQR or Q3+3*IQR) are indicated with a star.

Fig 1 shows the severity of depression symptoms for the relationship group and the heartbreak group. MDI total scores ranged between 2 and 22 (*Mdn* = 7.00, *IQR* = 4.75–10.25) in the relationship group. 97.8% were found to have a MDI score below 21, corresponding to an absence of depression. 2.2% had depression symptoms corresponding to mild depression. MDI scores ranged between 1 and 45 (*Mdn* = 9.00, *IQR* = 7.00–21.00) in the heartbreak group. 12.7% reported depression symptoms corresponding to mild depression. 1.4% and 12.7% reported symptoms corresponding to respectively moderate and severe depression. In total, 26.8% reported symptoms corresponding to mild, moderate or severe depression. MDI total scores were higher in the heartbreak group compared to the relationship group (*U* = 1042.00, *Z* = -3.31, *p* = .001, *r* = -.31). No gender differences were found (*U* = 213.50, *Z* = -1.13, *p* = .260, *r* = -.17) between the males (*Mdn* = 6.00, *IQR* = 4.00–8.00) and females (*Mdn* = 7.00, *IQR* = 5.00–14.00) in the relationship group. MDI scores differed (*U* = 380.00, *Z* = -2.85, *p* = .004, *r* = -.34) between heartbroken males (*Mdn* = 7.00, *IQR* = 4.50–14.00) and heartbroken females (*Mdn* = 15.50, *IQR* = 7.75–25.00). S3 Table shows the median and interquartile range for the individual items for the two groups. With regard to the core symptoms of depression, the heartbreak group scored higher on the item ‘‘feeling sad or low in spirits” and the item ‘‘loss of interest in daily activities” (*p* = .001 and *p* = .013), while the item ‘‘lack of energy and strength” did not differ between the two groups (*p* = .218). Concerning the accompanying symptoms of depression, the items ‘‘feeling less self-confident”, ‘‘the feeling that life was not worth living”, ‘‘concentration difficulties”, ‘‘feeling restless/listless” and ‘‘sleeping difficulties” differed significantly between the two groups (all higher in the heartbreak group, *p* = .019, *p* = .002, *p* = .019, *p* < .001, and *p* = .004, respectively). No differences were found regarding the items ‘‘feelings of guilt” and ‘‘decreased/increased appetite” (*p* = .112 and *p* = .151).

### Characterization of heartbreak

To characterize the heartbreak group, a PCA-varimax was performed on the questionnaire battery. Subsequently, the relation with the depression scores was investigated.

### Components extraction

Parallel analysis revealed two components with corresponding eigenvalues greater than the concomitant eigenvalue calculated for a random dataset, explaining respectively 45.8% and 13.8% of the variance.

### Component loadings and interpretation

Table 1 shows the component loadings for the included variables for the two components. Additionally, the 95% CIs of the component loadings are shown. Given that the variables ‘‘feeling betrayed”, ‘‘feeling rejected”, ‘‘feeling angry”, ‘‘unexpectedness breakup” and ‘‘ICG” load highly on component 1 (95% CI does not straddle zero), this component was interpreted as ‘‘sudden loss”. The variables ‘‘feeling hopeful” and ‘‘PANAS positive” load strongest (inversely) on component 2 and have 95% CIs that do not contain zero. Therefore, this component was interpreted as ‘‘lack of positive affect”.

**Table 1:** Component loadings and 95% CIs for the 19 variables that were included into the PCA-varimax.

	Component 1	Component 2
1. Unexpectedness breakup	.78 [.67, .87]	-.13 [-.27, .15]
2. Feeling rejected	.88 [.79, .92]	.12 [.03, .36]
3. Feeling betrayed	.89 [.81, .93]	-.11 [-.19, .15]
4. Feeling angry	.84 [.69, .92]	-.01 [-.11, .24]
5. Feeling relieved	-.47 [-.61, -.23]	-.57 [-.74, -.42]
6. Feeling sad	.67 [.48, .76]	.51 [.42, .69]
7. Feeling disappointed	.73 [.56, .81]	.40 [.28, .64]
8. Feeling independent	.04 [-.20, .34]	-.57 [-.72, -.35]
9. Feeling alone	.48 [.25, .60]	.62 [.46, .80]
10. Feeling hopeful	-.10 [-.22, .13]	-.83 [-.89, -.73]
11. Ruminating thoughts	.57 [.38, .65]	.70 [.63, .82]
12. Intrusive thoughts	.52 [.30, .62]	.58 [.45, .75]
13. In love with ex-partner	.50 [.24, .65]	.50 [.33, .72]
14. Affection for ex-partner	.02 [-.29, .30]	.60 [.37, .75]
15. ICG	.78 [.63, .83]	.49 [.43, .66]
16. PANAS positive	.10 [-.05, .34]	-.76 [-.85, -.57]
17. PANAS negative	.61 [.38, .75]	.42 [.26, .66]
18. PRQC	.62 [.38, .74]	.25 [.06, .55]
19. Hurt proneness	.13 [-.17, .44]	.26 [-.11, .56]

### Association between the components and time since breakup and relationship duration

No significant correlation between time since breakup and each of the two components was found (‘‘sudden loss”: *r*_*s*_ = .06, *p* = .600, “lack of positive affect”: *r*_*s*_ = -.22, *p* = .071). Relationship duration correlated significantly with the ‘‘lack of positive affect” component (*r*_*s*_ = .25, *p* = .039) and did not correlate significantly with the ‘‘sudden loss” component (*r*_*s*_ = -.07, *p* = .559).

### Association between the components and depression scores

**Fig 2:**
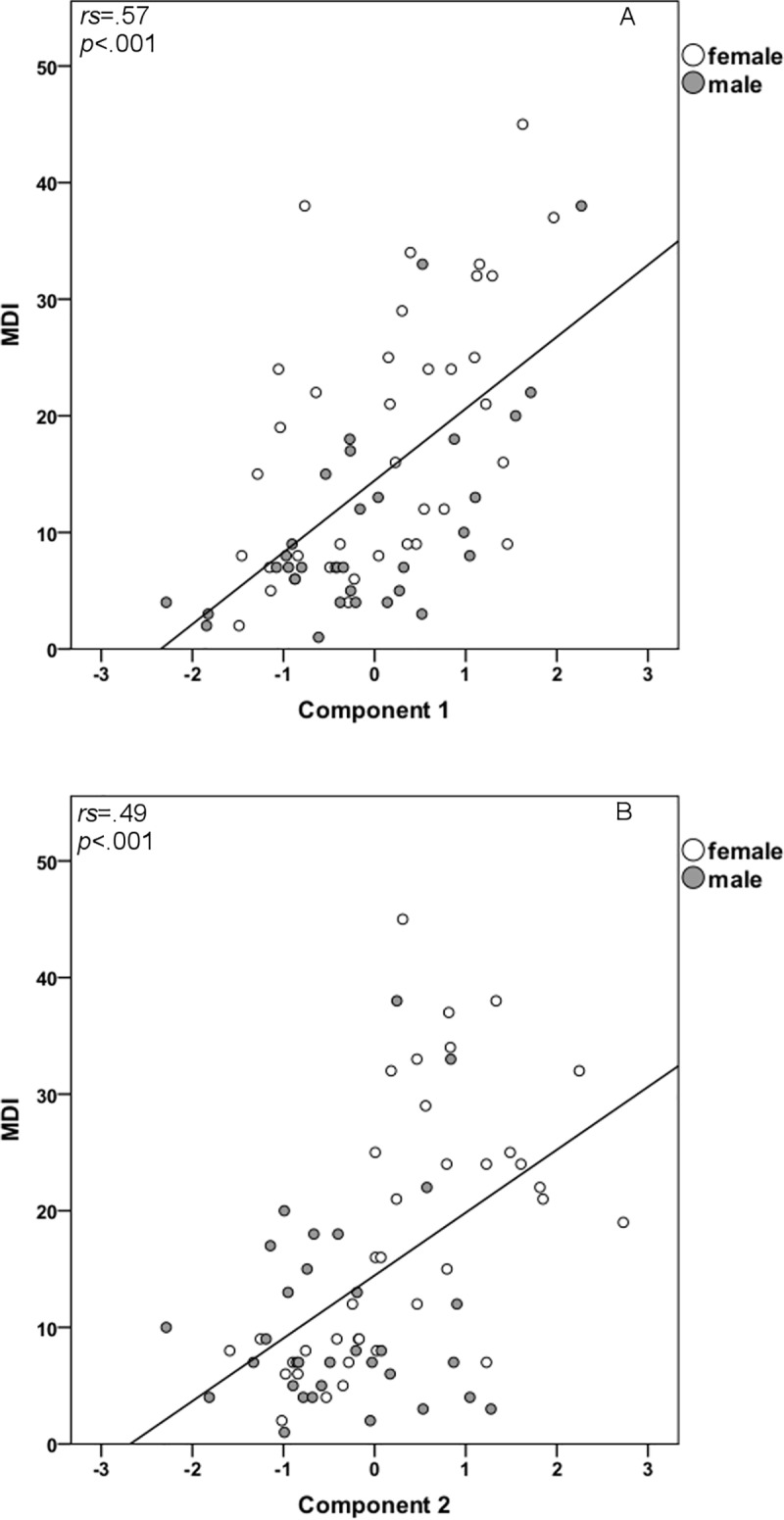
Relationship between each of the components and MDI scores. (A) Relationship between the ‘‘sudden loss” component and MDI. (B) Relationship between the ‘‘lack of positive affect” component and MDI.

Positive correlations between the component scores belonging to the two extracted components and depression scores were prevalent (*r*_*s*_ = .57, *p* < .001 and *r*_*s*_ *=* .49, *p* < .001 for the ‘‘sudden loss” component and the ‘‘lack of positive affect” component, respectively). The scatterplot between the ‘‘sudden loss” component and MDI and between the ‘‘lack of positive affect” component and MDI are shown in Fig 2A and Fig 2B, respectively.

### Gender effects components

Gender differences with regard to the ‘‘lack of positive affect” component were found: heartbroken females had higher component scores (*t*(67) = 2.95, *p* = .004, *r* = .34). Component scores belonging to the ‘‘sudden loss” component did not differ between the genders (*t*(67) = .88, *p* = .385, *r* = .11). MDI scores correlated positively with both components (see above). However, the MDI score showed a gender effect as well (see above). Therefore, correlations between MDI scores and each of the two components were examined for men and women separately. For heartbroken females, highly significant correlations were found for both ‘‘sudden loss” and ‘‘lack of positive affect” (*r*_*s*_ = .57, *p* < .001 and *r*_*s*_ *=* .70, *p* < .001 respectively). Heartbroken males showed a partially different result: a significant correlation between MDI scores and the ‘‘sudden loss” component was prevalent (*r*_*s*_ = .55, *p* = .001). In contrast, the ‘‘lack of positive affect” component did not correlate significantly with MDI scores (*r*_*s*_ = -.01, *p* = .951).

## Discussion

In the present study, we primarily aimed to investigate: 1) whether individuals with a recent romantic relationship breakup demonstrate symptoms of depression, 2) how to describe heartbreak characteristics based on data from a comprehensive questionnaire battery, and 3) whether this description can capture severity of depression symptoms. Secondary, we were interested in gender differences with regard to the above study objectives.

In accordance with our expectations, severity of depression symptoms was found to be higher in the heartbreak group compared to the reference group, i.e. subjects in a romantic relationship. MDI total scores as well as individual items, including core symptoms of depression, were elevated. However, median MDI scores of the heartbreak group fell within the range of absence of depression as defined by Bech et al.[20]. Nonetheless, 26.8% and 14.1% of the heartbroken subjects reported severity of depression symptoms corresponding to respectively mild to severe depression and moderate to severe depression. In contrast, only one subject reported symptoms corresponding to (mild) depression in the relationship group. In a study by Forsell et al.[25], a mean MDI score of 8.8 (95% CI 8.6–9.0) was found in a large sample of men and women drawn from the general population. Note that even in this general population, 8.0% reported symptoms corresponding to moderate or severe depression[25] (compared to the 14.1% found in this study). Thus, we consider the heartbreak group as a good population to study a depression-like state in otherwise healthy individuals.

We described heartbreak by two principal components. Feelings of betray, rejection and anger, unexpectedness of the breakup and symptoms of complicated grief contributed substantially to the first component that was therefore interpreted as ‘‘sudden loss”. Feeling hopeful after the breakup and current positive affect (i.e. the ability to experience positive emotions) contributed largely (inversely) to the second component that was consequently interpreted as ‘‘lack of positive affect”. The finding that the feeling of being betrayed is an important parameter of heartbreak is consistent with the study of Field et al.[3]. Moreover, our findings show similarities with a retrospective study concerning emotions following a relationship dissolution by Barbara and Dion[26]. In that study, a component labeled as ‘‘negative emotions” was extracted and rejection and anger were found to be important variables for that specific component[26]. This is in accordance with the high loadings of the variables ‘‘feeling rejected” and ‘‘feeling angry” on the ‘‘sudden loss” component as found in our study.

Within the heartbreak group, both components correlated highly with depression scores. The ‘‘lack of positive affect” component is primarily defined by positive affect scores, as measured with the PANAS. This is in accordance with a study by Crawford and Henry[27] in which positive affect was found to be specifically related to depression scores in a large sample of men and women drawn from the general population. The ‘‘sudden loss” component also correlated highly with depression scores. This is consistent with literature regarding grief. For example, Keyes et al.[28] found associations between the experience of an unexpected death of a loved one and prevalence of psychiatric problems including clinical depression.

As expected, heartbroken females reported higher depression scores than heartbroken males in our study. This cannot be explained by general gender differences, given that the depression scores of the men and women of the relationship group did not differ, and therefore seems to be breakup-related. Among the heartbroken males examined separately, no association between the ‘‘lack of positive affect” component and severity of depression symptoms was found. Additionally, women had higher scores on the ‘‘lack of positive affect” component than men. Tentatively, these findings suggest that men are less likely to demonstrate and/or report reduced abilities experiencing positive emotions during a period of stress than women and this possibly relates to the well-known differential depression rates among the genders.

### Limitations

By conducting the present study, detailed knowledge about behavioral and psychological consequences of a recent romantic relationship breakup and its association with symptoms of depression was acquired. A potential weakness of our study is that differences in recruitment strategy and pre-selection prior to inclusion between the genders could have influenced our findings. This makes it difficult to draw strong conclusions about effects of gender. Nevertheless, gender-specific application rates can be considered a finding as well in our opinion. Another possible weakness is that already having a new romantic partner was not an exclusion criterion in our study and in our sample five of the 71 subjects reported to have found a new partner on the day of the experiment. One could argue that this will reduce sadness and mood problems associated with a breakup. However, excluding those subjects from our dataset did not change either group-level differences regarding depression scores or the strength of the correlation between the components and depression scores noticeably (data can be found in S1 Appendix). Therefore, possible effects of having a new romantic partner on our results were considered minimal. Perhaps, finding a new partner cannot diminish breakup-related effects within this limited period of time.

## Conclusions

In the present study, we investigated whether the breakup of a romantic relationship can be used as an experimental model to study a depression-like state. We demonstrated an increased range of depression scores among our sample of individuals who recently have experienced a relationship breakup. Furthermore, our results show that the effects of experiencing a relationship breakup can be captured with two descriptors: “sudden loss” and “lack of positive affect”. Both were associated with (severity of) depression (-like) symptoms. Nota bene, this association was gender-dependent. Therefore, we propose that this life-event is a viable experimental model to investigate symptoms of depression in individuals without a psychiatric disorder. This paves the way to investigate the involvement of stress in the transition from healthy-to depressive behavior. Consequently, further longitudinal research using this model could provide new insights into individual-specific coping and vulnerability factors contributing to the development of depression symptoms during a period of stress.

## Supporting information

S1 Table Cronbach’s alpha scores questionnaire battery. (DOCX)

S2 Table Additional background information of the heartbreak group and the relationship group. Values are shown as percentage or median (Q1-Q3) for respectively categorical variables and numerical variables.
(DOCX)

S3 Table Individual MDI items for the relationship group and the heartbreak group. Values are shown as median (Q1-Q3).
(DOCX)

S1 Appendix Group-level differences depression scores and correlation components and depression scores after excluding subjects with a new partner. (DOCX)
