# Supplementary material for: Romantic relationship breakup: An experimental model to study effects of stress on depression (-like) symptoms
Source: PLoS One. 2019 May 31;14(5):e0217320. doi: 10.1371/journal.pone.0217320 (PMC6544239; doi:10.1371/journal.pone.0217320)
Supplement: S1 Table — (DOCX) [file pone.0217320.s001.docx]

| **Questionnaire** | **Cronbach’s alpha score relationship group (*N*=46)** | **Cronbach’s alpha score heartbreak group (*N*=71)** |
| --- | --- | --- |
| **MDI** | .719 | .926 |
| **ICG** |  | .939 |
| **PANAS positive** | .703 | .876 |
| **PANAS negative** | .794 | .907 |
| **PRQC** | .876 | .816 |
| **Hurt-Proneness Scale** | .795 | .752 |
