# Supplementary material for: Romantic relationship breakup: An experimental model to study effects of stress on depression (-like) symptoms
Source: PLoS One. 2019 May 31;14(5):e0217320. doi: 10.1371/journal.pone.0217320 (PMC6544239; doi:10.1371/journal.pone.0217320)
Supplement: S2 Table — Values are shown as percentage or median (Q1-Q3) for respectively categorical variables and numerical variables. (DOCX) [file pone.0217320.s002.docx]

|  |  | **Relationship (*N*=46)** | **Heartbreak (*N*=71)** |
| --- | --- | --- | --- |
| **Education** | high school | 82.6 | 60.6 |
|  | MBO | 4.3 | 7.0 |
|  | HBO | 6.5 | 11.3 |
|  | university | 6.5 | 21.1 |
| **Occupation** | student | 80.4 | 73.2 |
|  | student and working | 17.4 | 18.3 |
|  | working | 2.2 | 8.5 |
| **Marital status parents** | never married | 2.2 | 7.0 |
|  | happy marriage | 69.6 | 54.9 |
|  | unhappy marriage | 6.5 | 8.5 |
|  | divorced | 21.7 | 29.6 |
| **Family situation** | with siblings | 100.0 | 91.3 |
|  | without siblings | 0.0 | 8.7 |
| **Chronotype** | morning type | 30.4 | 9.9 |
|  | evening type | 43.5 | 64.8 |
|  | none of the options | 26.1 | 25.4 |
| **Lifestyle regularity** | regular lifestyle | 37.0 | 21.1 |
|  | irregular lifestyle | 17.4 | 47.9 |
|  | varies across time | 45.7 | 31.0 |
| **Number of previous relationships** | 0 | 52.2 | 31.0 |
|  | 1-2 | 39.1 | 60.6 |
|  | 3-4 | 8.7 | 5.6 |
|  | >4 | 0 | 2.8 |
| **Number of previous heartbreak** |  | 1.00 (0.00-1.00) | 1.00 (0.00-2.00) |
