# Supplementary material for: Romantic relationship breakup: An experimental model to study effects of stress on depression (-like) symptoms
Source: PLoS One. 2019 May 31;14(5):e0217320. doi: 10.1371/journal.pone.0217320 (PMC6544239; doi:10.1371/journal.pone.0217320)
Supplement: S3 Table — Values are shown as median (Q1-Q3). (DOCX) [file pone.0217320.s003.docx]

| **MDI item** | **Relationship (*N*=46)** | **Heartbreak (*N*=71)** |
| --- | --- | --- |
| **1.Feeling sad or low in spirits** | 2.00 (1.75-2.00) | 2.00 (2.00-4.00) |
| **2.Loss of interest in daily activities** | 2.00 (1.00-2.00) | 2.00 (1.00-4.00) |
| **3.Lack of energy and strength** | 2.00 (2.00-3.00) | 2.00 (2.00-4.00) |
| **4.Feeling less self-confident** | 2.00 (1.00-2.00) | 2.00 (1.00-3.00) |
| **5. Feelings of guilt** | 1.00 (1.00-2.00) | 2.00 (1.00-2.00) |
| **6.The feeling that life was not worth living** | 1.00 (1.00-1.00) | 1.00 (1.00-2.00) |
| **7.Concentration difficulties** | 2.00 (1.00-2.00) | 2.00 (1.00-3.00) |
| **8.Feeling restless/listless** | 2.00 (1.00-2.00) | 3.00 (2.00-4.00) |
| **9.Sleeping difficulties** | 1.00 (1.00-2.00) | 2.00 (1.00-3.00) |
| **10.Decreased/increased appetite** | 2.00 (1.00-2.00) | 2.00 (1.00-3.00) |
