# Supplementary material for: Romantic relationship breakup: An experimental model to study effects of stress on depression (-like) symptoms
Source: PLoS One. 2019 May 31;14(5):e0217320. doi: 10.1371/journal.pone.0217320 (PMC6544239; doi:10.1371/journal.pone.0217320)
Supplement: S1 Appendix — (DOCX) [file pone.0217320.s004.docx]

MDI total scores were higher in the heartbreak group (*N*=66) compared to the relationship group (*N*=46) (*U*=959.00, *Z*=-3.32, *p*=.001, *r*=-0.31).

Positive correlations between the component scores belonging to the two components and MDI total scores were prevalent (*r_s_*=.57, *p* <.001 and r_s_=.49, *p* <.001 for the ‘’sudden loss’’ component and the ‘’lack of positive affect’’ component, respectively).

So, excluding those five subjects who reported to have found a new romantic partner from our dataset did not change either group-level differences regarding MDI scores or the strength of the correlation between the components and MDI scores noticeably.
